# Analysis and modeling of time-course gene-expression profiles from nanomaterial-exposed primary human epidermal keratinocytes

**DOI:** 10.1186/1471-2105-10-S11-S10

**Published:** 2009-10-08

**Authors:** Amin Zollanvari, Mary Jane Cunningham, Ulisses Braga-Neto, Edward R Dougherty

**Affiliations:** 1 Department of Electrical and Computer Engineering, Texas A&M University, College Station, TX, USA; 2 Nanomics Biosciences, Cary, NC, USA; 3 Department of Pathology, University of Texas MD Anderson Cancer Center, Houston, TX, USA; 4 Computational Biology Division, Translational Genomics Research Institute, Phoenix, AZ, USA; 5 Previous affiliation: The Houston Advanced Research Center, The Woodlands, TX, USA

## Abstract

**Background:**

Nanomaterials are being manufactured on a commercial scale for use in medical, diagnostic, energy, component and communications industries. However, concerns over the safety of engineered nanomaterials have surfaced. Humans can be exposed to nanomaterials in different ways such as inhalation or exposure through the integumentary system.

**Results:**

The interactions of engineered nanomaterials with primary human cells was investigated, using a systems biology approach combining gene expression microarray profiling with dynamic experimental parameters. In this experiment, primary human epidermal keratinocytes cells were exposed to several low-micron to nano-scale materials, and gene expression was profiled over both time and dose to compile a comprehensive picture of nanomaterial-cellular interactions. Very few gene-expression studies so far have dealt with both time and dose response simultaneously. Here, we propose different approaches to this kind of analysis. First, we used heat maps and multi-dimensional scaling (MDS) plots to visualize the dose response of nanomaterials over time. Then, in order to find out the most common patterns in gene-expression profiles, we used self-organizing maps (SOM) combined with two different criteria to determine the number of clusters. The consistency of SOM results is discussed in context of the information derived from the MDS plots. Finally, in order to identify the genes that have significantly different responses among different levels of dose of each treatment while accounting for the effect of time at the same time, we used a two-way ANOVA model, in connection with Tukey's additivity test and the Box-Cox transformation. The results are discussed in the context of the cellular responses of engineered nanomaterials.

**Conclusion:**

The analysis presented here lead to interesting and complementary conclusions about the response across time of human epidermal keratinocytes after exposure to nanomaterials. For example, we observed that gene expression for most treatments become closer to the expression of the baseline cultures as time proceeds. The genes found to be differentially-expressed are involved in a number of cellular processes, including regulation of transcription and translation, protein localization, transport, cell cycle progression, cell migration, cytoskeletal reorganization, signal transduction, and development.

## Background

Nanomaterials are being manufactured on a commercial scale for use in medical, diagnostic, energy, component and communications industries [1,2]. Engineered nanomaterials range considerably in their physicochemical properties making them more desirable than their micro- and macro-counterparts due to, for example, their increased surface area, tensile strength, tunability, etc. [3]. From limited early reports, concerns over the safety of engineered nanomaterials have surfaced [4,5]. Humans can be exposed to nanomaterials in different ways such as inhalation or exposure through the integumentary system. However, the skin is a unique organ in the body not only because it gives the body such a large surface area for exposure but also because of the avascular property of epidermis, in which particles can reside without being removed by phagocytosis [6].

Gene expression microarrays have become a tool to investigate the interactions of biological systems by observing the simultaneous activities of tens of thousands of genes. Over recent years, this tool has been applied to toxicology forming a new discipline, toxicogenomics [7,8]. Microarrays have most recently been a tool used by pharmaceutical drug discovery and development to screen for efficacy and adverse effects thereby prioritizing drug candidates and redeveloping ones which show off-target toxicities [9-11].

The approach described here combines a global screening technology, gene expression microarray profiling, with systems biology, to investigate the interactions of engineered nanomaterials with primary human cells. The biological and cellular system is perturbed and reiteratively sampled over both time and dose to compile a more comprehensive picture of nanomaterial-cellular interactions. From over 100 papers which were reviewed by the authors in [12], only 3 papers have dealt with the effect of concentration plus time while the remaining papers dealt only with the parameter of time. Initial studies that were published previously focused on reporting significantly-expressed genes and using clustering methods to identify similarities and differences between expression profiles [13-15]. In addition, the study cited in [12] was the only previously combined study investigating time-course and dosage-effect simultaneously, while the initial 3 cited studies investigated time-effect and dosage effect separately.

In the present study, we propose different approaches to this kind of analysis. We considered the gene expression of primary human epidermal keratinocytes, under exposure to the following low-micron to nano-scale materials: carbonyl iron (FC), carbon black (CB) silica (SiO_2_) and single-walled carbon nanotubes (SWNT), at noncytotoxic and cytotoxic doses for each. The nanomaterials used, except for the carbonyl iron (FC) and SWNT, are not intended for medical use. These materials are currently being used in construction materials, consumer goods, and communications and IT applications. The FC nanoparticles were used as a negative control compound and have been approved for use by the FDA as a pharmaceutical carrier formulation. The single-walled carbon nanotubes are being developed for medical applications (e.g. drug carriers or medical imaging compounds) only after being functionalized with other components. We remark here that the goal of the experimental design was not to study particle size or penetration effects. It was to study whether there was an overall interaction with the nanomaterials. In particular, the cytotoxic dose (i.e. high dose) used with the carbonyl iron was due to its toxic effect of an overload of iron on the cells.

In the approach discussed here, we first used heat maps and multi-dimensional scaling (MDS) plots to visualize the dose response of nanomaterials over time. Then, in order to find out the most common patterns in gene-expression profiles, we used self-organizing maps (SOM) combined with two different criteria to determine the number of clusters. The consistency of the SOM results is discussed in context of the results from the MDS plots. Finally, in order to identify the genes that have significantly different responses among the different dose levels while simultaneously accounting for the effect of time, we have used a two-way ANOVA model, in connection with Tukey's additivity test and the Box-Cox transformation. These analysis results are discussed in the context of the biological and cellular interactions of the engineered nanomaterials.

## Methods

### Biological experimental design

Primary human epidermal keratinocytes (HEK, Cascade Biologics) were cultured with serum-free defined media at 5% CO_2 _and 37°C. The HEK were exposed to the following low-micron to nano-scale materials: carbonyl iron (FC), carbon black (CB) silica (SiO_2_) and single-walled carbon nanotubes (SWNT). FC was supplied by ISP Technologies with a mean particle size of 5.8 μm, CB was supplied by Degussa with a mean particle size of 17 nm, and SiO_2 _was supplied by US Silica with a mean particle size of 1.6 μm. SWNT was prepared by SouthWest Nanotechnologies with a mean diameter of 0.8 nm. The purity of SWNT was checked, and it was found that all heavy metal contamination was very low, less than 1%. The nanotubes were acid-purified from the raw manufactured intermediate. The average length of the SWNT nanotubes was 960 nm; this is the only nanomaterial among the ones considered here that is not spherical. From previous viability assays [13,15], two different doses were selected for the exposure of each nanomaterial – a noncytotoxic dose and a cytotoxic dose. The cytotoxic dose was extrapolated from the viability curve of each substance where 50% of the cells were still viable. The noncytotoxic and cytotoxic doses for each nanomaterial were found to be, respectively, 0.03 and 1 (FC), 0.01 and 0.5 (CB), 0.1 and 1 (SiO_2_), and 0.001 and 1 (SWNT), all concentrations being expressed in mg/ml. The cells were exposed to FC, CB, SiO_2_, and SWNT and harvested at 2, 4, 6, 8, 12, 18, and 24 hours after exposure. Cells cultured under the same conditions and not exposed to any of the nanomaterials were harvested at the same time points for a time-matched control baseline.

The cells were trysinized and cell counts taken. The cells were then collected by centrifugation, snap frozen in liquid nitrogen, and stored at -80°C. The frozen pellets were shipped to GenUs Biosystems for isolation of the total RNA and processing of the gene expression microarrays. Total RNA was isolated from the cell pellets, reverse-transcribed to biotinylated cRNAs and hybridized onto 10 K human gene expression microarrays (GE Healthcare). The corresponding cRNA for each biological sample was hybridized to triplicate microarrays. The arrays were rinsed, dried, scanned and image analyzed and the flat files returned to the Houston Advanced Research Center.

### Data analysis and preprocessing

The microarray flat files contained the quantitative expression values for all probes (positive controls, negative controls, fiducial, and discovery) and all discovery probe values were assessed with a quality flag. Only those probes that have "DISCOVERY" and "G" (Good-quality) tags across all time points and dose levels of all treatments were considered for further analysis to guarantee maximal reproducibility of the results. By using this criterion, the number of genes was reduced from 10,458 to 2,370. Then the average of the three replicate measurements was considered as the actual expression level of each gene at that time in its corresponding treatment.

### Heatmaps

The heatmaps are visualizations of hierarchical clustering [16] using "average" linkage and Pearson Correlation as the distance metric between expression levels of each gene after taking the log ratio of the data. It is worthy to mention that Pearson correlation is in fact the normalized correlation between values of two random variables that have had their mean subtracted from them. The dendrogram added to the left side of each heat map is obtained by Pearson correlation. The heatmaps were drawn by first subtracting the mean of each row from each data cell and then normalizing each row to obtain a variance of 1. Note that normalization was performed after taking the log ratio of the expression of each gene between the given treatment and the control baseline. Heatmaps obtained in this way reveal the underlying shape of expression pattern of genes better and are less affected by some large values which may be the result of noise. For more information on applying hierarchical clustering to gene expression patterns, see [16] and [17] and the references mentioned there.

### Multi-dimensional Scaling (MDS)

Multidimensional Scaling (MDS) is a method to project high dimensional data onto a lower dimension while maintaining the approximate distances between data points [21]. The accuracy of the representation is measured by the "stress." A stress of 10% is usually considered good.

First, the data are collected by taking the log of the expression values of genes in each treatment and the baseline. Then at each time point, a matrix of distances between the gene-expression profiles corresponding to each treatment and the baseline is created, where the distance is calculated as 1 – the Pearson correlation between the profiles. Finally, MDS is performed based on this matrix, in order to obtain a low-dimensional representation of the gene-expression profiles and the distance among them.

### Self-Organizing Maps (SOM)

SOM [15] is a powerful method for clustering. After log-normalizing the data as explained above, this method was employed in two ways.

First, the underlying hidden number of clusters of the data was estimated using two different criteria the silhouette criterion [18] and the CLEST criterion [19]. In [19], it has been shown that the CLEST criterion is the stronger tool to find number of clusters. However, from the comparison of the CLEST and the silhouette criteria in [19], it is inferred that whenever the true number of clusters has been equal to 2, the silhouette criterion is the same as the CLEST or has done a better job than the CLEST criterion, while for greater number of clusters, the CLEST performs better. Therefore, the criterion that we have used to determine the number of clusters is a combination of the two criteria. First, the silhouette criterion is implemented to determine the number of clusters. If the number of clusters for any treatment is equal to 2, and if the silhouette width for this number of clusters is fairly close to 1 (e.g. greater than 0.6) then we assigned 2 as the optimal number of clusters. Otherwise, the CLEST criterion is implemented to determine the number of clusters.

In the second way of implementing SOM, larger numbers of clusters than the previous approach were tried, as a means of obtaining clusters that show tighter expression patterns than in the first approach.

### ANOVA model

A two-way ANOVA model was used to identify genes for which there are significant differences in the response to the various dose levels, and have them ranked by the *p*-values of dose main effect. The general procedure was as follows:

0 – For each gene, compile the data in a two-dimensional array consisting of the expression values from the three levels of dose (untreated, low and high) and the eight time points. Use Tukey's additivity test for two-way ANOVA (one observation per cell) to see if there is interaction between time and dose for the given gene. If there is no interaction go to step 2.

1 – If there is significant interaction between these two factors, try to remove the interaction by using the Box-Cox transformation [20]. The removal of interaction can be tested by Tukey's additivity test for transformed values corresponding to each parameter of the Box-Cox transformation. We have chosen this parameter to vary between -2 to 2 with 0.1 spacing. If the interaction is removed, use these as the new data for the given gene. If the interaction could not be removed, set the p-value of dose main effect to NA and go to step 0 to start the process for the next gene.

2 – Use two-way ANOVA to find whether there are significant differences among the levels of the dose and time factor. If there is not any significant difference on any factor, go to step 4.

3 – Use Tukey's HSD post-hoc test to determine between which levels of either factor there are significant differences and record them. Since Tukey's HSD test is conservative, in rare cases the test cannot find any significant difference between levels.

4 – Save the p-value for the difference in dose main effect for this gene and go to step 0 to start the process for the next gene.

At the end of this process, sort all genes with respect to p-values on dose main effect. The lower the p-value is for a gene, the stronger the evidence is that there are significant differences in response among the various levels of dose for that gene.

## Results

### A. Heatmap plots

**Figure 1:**
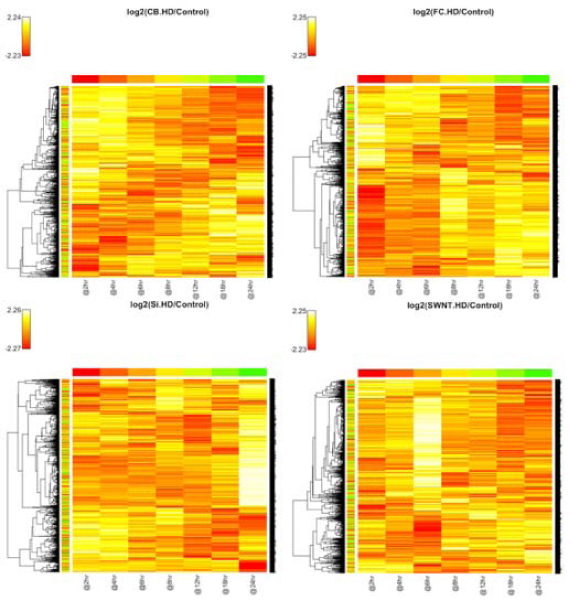
**Heat map and Hierarchical Clustering for the high-dose treatments**.

**Figure 2:**
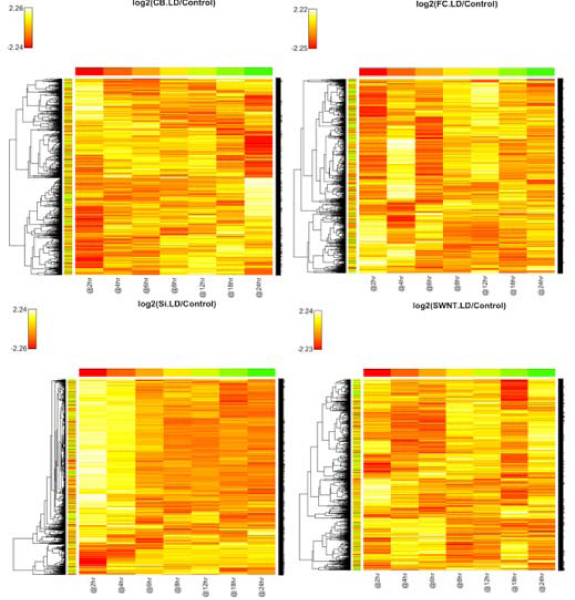
**Heat map and Hierarchical Clustering for the low-dose treatments**.

For the heatmaps, we consider the log ratio between the gene expression in one of the treatment time-courses and the corresponding gene expression in the baseline time-course, for each gene and each time point, and plot the log ratio for each gene (rows) versus time point (columns). There are 8 heatmaps, corresponding to each of the 8 treatments (including low and high dose), which are displayed in Figures 1 and 2. In these plots, the closer the color of a cell is to white (positive log ratio, up-regulation) and red (negative log ratio, down-regulation), the more difference there is between the expression value of that gene between the given treatment and the baseline at the particular time point; by contrast, orange indicates an absence of differential expression with respect to the baseline. Considering, for instance, the SiO_2 _LD (low dose) heatmap, we can see that differential expression tends to occur early in the time course (between 0 and 4 hr) and tends to disappear with time. It can also be observed in this heatmap that the genes fall in basically two clusters with respect to their time-course expression: a majority of genes that are up-regulated early, and a minority that is down-regulated early and slightly up-regulated later in the time course.

### B. Multi-Dimensional Scaling (MDS) plots

**Figure 3:**
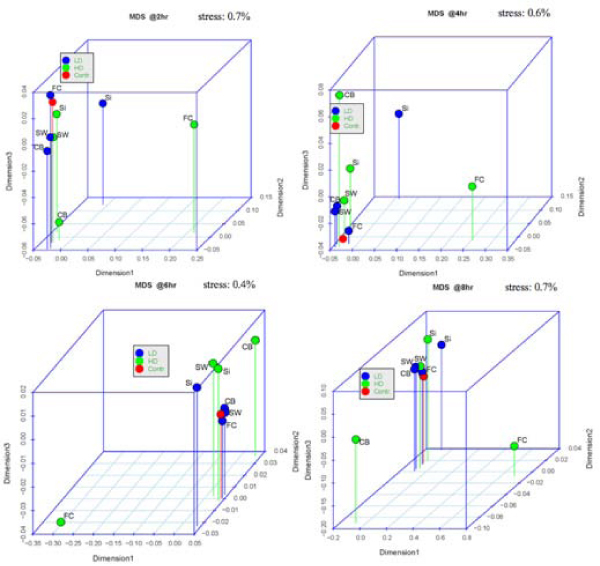
**Sequence of MDS plot from time 2 hr to 8 hr**.

**Figure 4:**
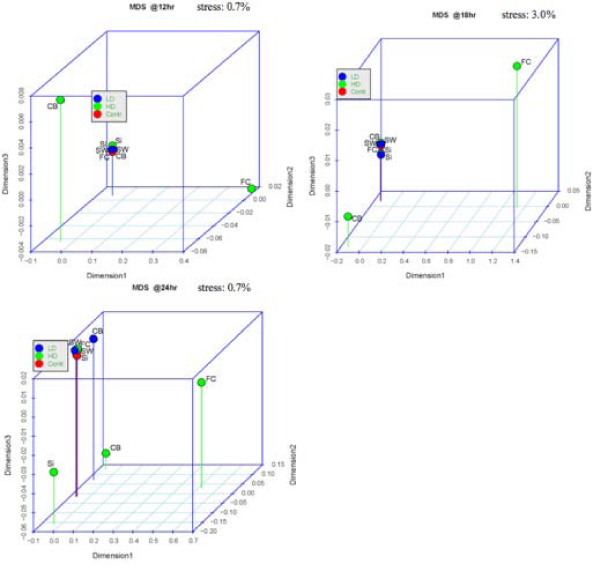
**Sequence of MDS plot from time 12 hr to 24 hr**.

For the MDS plots, we consider the log of the absolute gene expression for each of the treatments and the baseline (no ratio is taken). There are 7 plots, one for each time point, which are displayed in Figures 3 and 4. In these plots, the closer two points are to each other, the more similar their gene expression profiles are, according to the Pearson correlation (see Methods section). Considering, for instance, the plot at time 18 hrs, it can be easily seen that most of the treatments show similar gene expression to the baseline at this time (i.e., a weak transcriptional response), while the CB HD (high dose) and FC HD treatments present a stronger response. This is clear by the fact that all the treatments are concentrated in one point except CB HD and FC HD. In fact, one observes that all treatments tend to become closer to the baseline as time proceeds, except for CB HD and FC HD. This shows that these two treatments, generally, have the most effect on changing gene expression. The same fact can also be observed in the heatmaps of Figures 1 and 2.

### C. Self-Organizing Maps (SOM)

Here, we again consider the log ratio between the gene expression in one of the treatment time-courses and the corresponding gene expression in the baseline time-course, for each gene and each time point. Table 1 shows the optimal number of clusters for each treatment that were obtained by using a combination of the silhouette and CLEST criteria, as described in the Methods section. It is interesting to compare these numbers with what is deduced from visualizing the heatmaps in Figures 1 and 2. For example, Table 1 says that the number of clusters in SiO_2 _LD and CB HD is 2 and 4, respectively. This fact is consistent with what one may infer from Figures 1 and 2.

**Table 1:** Treatments and the their determined optimal number of clusters.

Treatment	Number of Clusters
CB HD	4
CB LD	5
FC HD	2
FC LD	3
Sio2 HD	10
Sio2 LD	2
SWNT HD	3
SWNT LD	5

**Figure 5:**
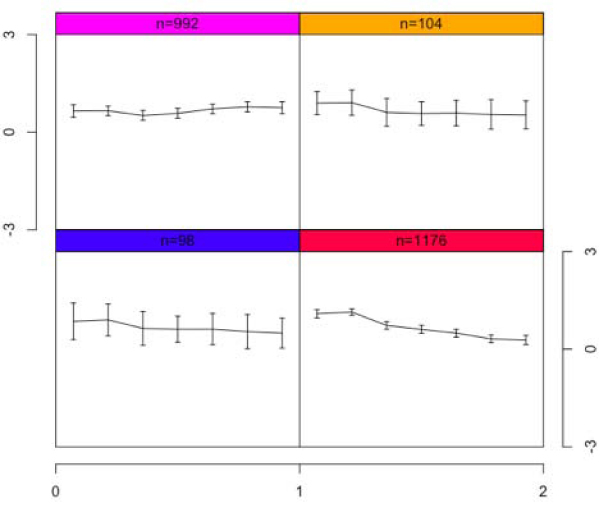
**SOM clusters for a 2 × 2 unit plane for CB HD**.

**Figure 6:**
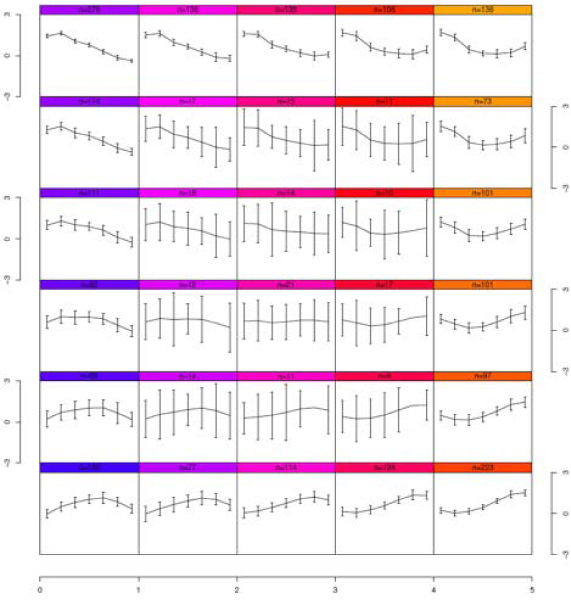
**SOM clusters for a 5 × 6 unit plane for CB HD**.

Figure 5 shows the average expression values of genes in each cluster by performing SOM on the CB HD treatment (the plots for the other treatments, along with the list of genes in each cluster, can be found in the supplementary information). In agreement with the fact that the optimal number of clusters in this treatment was determined to be four, we employed 2 × 2 SOM to find four clusters. This can be contrasted with Figure 6, which shows the result for the same treatment but now using 5 × 6 SOM to find 30 clusters. Obviously, as there are fewer genes in each cluster, we obtain tighter gene expression patterns (smaller standard deviations) in each cluster.

### D. ANOVA model

The two-way ANOVA model that we consider has as factors time and dose, where the former has 3 levels (baseline, low dose and high dose) and the latter has 7 levels, corresponding to the seven time points in the experimental design. Since we are interested in the dose main effect on a given gene, we attempt to remove interaction of dose with time. After determining those genes affected significantly by the dose main effect, it is worthy to investigate between which specific levels there is a difference, which is accomplished by the Tukey's HSD post-hoc test (see the Methods section for a detailed description of the procedure that was employed).

Table 2 show the top 40 gene IDs that respond to the CB HD treatment, out of the original list of 2370 genes (the tables for the other treatments can be found in the supplementary information). The genes were ranked in ascending order of the *p*-value resulting from the ANOVA analysis, which means that the genes at the top are expected to show stronger responses to the treatment. In addition, the last three columns of Table 2 show where the expression differences lie, according to Tukey's HSD test; "1" indicates a significant difference.

**Table 2:** Genes showing significant difference among various level of dose in treatment CB

Rank	Acc No.	Entrez Gene ID	Gene Name	HD-LD	HD-Control	LD-Control
1	AB023163	23390	ZDHHC17	1	1	1
2	NM_033495	90293	KLHL13	1	1	1
3	AL390214	7204	TRIO	1	1	1
4	BF057080	--	ENPP1	1	1	1
5	NM_002292	3913	LAMB2	1	1	0
6	NM_004231	9296	ATP6V1F	1	1	1
7	NM_004524	3993	LLGL2	1	1	1
8	U91543	1107	CHD3	1	1	1
9	AF052159	201562	PTPLB	1	1	0
10	AL049471	--	ARID5B	1	1	0
11	NM_001461	2330	FMO5	1	1	1
12	NM_002135	3164	NR4A1	1	1	0
13	NM_002827	5770	PTP1B	1	1	1
14	NM_004517	3611	ILK	0	1	1
15	NM_014489	27315	FRAG1	1	1	1
16	AL049218	--	RNASEH2B	1	1	1
17	BC013306	6923	TCEB2	1	1	1
18	D38521	23198	PSME4	1	1	1
19	NM_001273	1108	CHD4	1	1	0
20	NM_003789	8717	TRADD	1	1	1
21	NM_005374	4355	MPP2	0	1	1
22	NM_007112	7059	THBS3	1	1	1
23	AF090094	--	OAZ1	0	1	1
24	NM_000575	3552	IL1A	1	1	1
25	NM_003187	6880	TAF9	1	1	1
26	NM_005309	2875	GPT	1	1	1
27	NM_006451	10605	PAIP1	1	1	0
28	NM_022365	64215	DNAJC1	1	1	1
29	AI435998	--	C17orf51	1	1	1
30	AW021631	--	---------	1	1	1
31	NM_004603	6804	STX1A	1	1	0
32	NM_005182	766	CA7	1	1	1
33	AA601902	--	MAPK8IP3	1	1	1
34	AL137484	221191	KLKBL4	1	1	1
35	NM_001271	1106	CHD2	1	1	1
36	NM_005536	3612	IMPA1	1	1	0
37	X58529	3507	IGHM	0	1	1
38	NM_014489	27315	FRAG1	1	1	1
39	NM_016574	1813	DRD2	1	1	1
40	NM_023111	2260	FGFR1	1	1	1

## Discussion

We comment here on the results obtained for the various treatments. The analysis resulted in several gene families or genes associated with the same cellular processes which responded significantly to the treatments. Very little toxicity overall was seen with any nanomaterial at the noncytotoxic dose except for silica, the positive control substance. Pathways for inflammation and irritation were identified; e.g., these pathways were observed for the silica at a noncytotoxic dose. At the cytotoxic doses, the most toxicity was seen with carbonyl iron – the explanation for this being the iron overload experienced by the cells. On the other hand, the majority of the genes found show significant differences at both the low and high dose levels. We observed that gene expression level becomes closer to basal level in later time points. This trend is seen with all gene expression profiling where the initial hours show the most activity [49,50].

Next we provide next detailed comments on the genes displayed in Table 2, which are associated with significant response to the CB treatment. Many of these genes are notable. Three different chromodomain-helicase-DNA binding protein genes are seen: CHD2, CHD3 and CHD4. These genes code for proteins which form a histone deacetylase complex involved in chromatin remodeling, an important transcriptional process [22]. Two different genes coding for proteins in the protein tyrosine phosphatase or protein tyrosine phosphatase-like families, PTP1B and PTPLB, are found to be highly significant. In addition, four genes associated with apoptosis are found. IL1A and TRADD are both involved in the NF-KappaB signaling pathways [23,24]. NR4A1 is an early response gene which codes for a nuclear orphan receptor and has an active role in cellular stress [25]. TAF9 is a TATA box-binding protein associated factor binds histones and is important in transcriptional regulation and cell viability [23-26]. Additionally, another protease, PSME4, which is related to the ubiquitin pathway, was found [27]. STX1A is a gene that may be involved Parkinson's disease as it translates to proteases located in brain tissue [28]. The gene PAIP1 is involved in the regulation of translation. PAIP1 is very similar to EIF4A (an eukaryotic initiation factor) which has a role in binding mRNA molecules to the ribosome. PAIP1 is active in mRNA turnover and their deadenylation and decay [29]. One gene, FRAG1, appears twice as two different FRAG1 probes were found to be significant within the top 40 genes.

Genes that show differential expression at both dose levels include MAPK8IP3, TRIO, ATP6V1F, and Hs. 656094 (gene index 2145). TRIO regulates the reorganization of the cytoskeleton and is essential for cell migration and cell growth [30]. ATP6V1F is active in transport mechanisms; it mediates the acidification of intracellular compartments essential to ion transport across cellular membranes [31,32]. MAPK8IP3 is involved in signal transduction by coding for a scaffold protein in the JNK signaling pathway [33]. It is activated by cytokines and environmental stresses. One gene (Hs. 656094, gene index 2145) is unannotated, being a transcribed locus that translates to a protein with no known functions.

Several genes show differential expression only with the high dose. These genes include IL1A, LAMB2, IMPA1, ARID5B, CHD4 and ARHGAP22. The genes ARHGAP22, ARID5B and CHD4 are involved in the regulation of transcription. ARHGAP22 codes for a rac GPTase active in signal transduction and seems to play an indirect role in cytoskeletal reorganization and cell motility [34]. ARID5B regulates transcription by binding to DNA and has important roles in development and differentiation [35]. CHD4 represses transcription by modulating the amount of protein at the centrosome during mitosis [36]. IL1A is involved in apoptosis as well as many roles in immune and inflammatory responses and hematopoiesis [37]. The last two genes, LAMB2 and IMPA1 are both involved in development. IMPA1 has an essential role in phosphatidylinositol pathways and LAMB2 codes for a laminin isoform which is involved in embryonic development and the organization of cells into tissues [38].

Table 3 breaks down where differences are found among the 40 genes on the top of list of each treatment. It can be seen from this table that CB is a treatment under which there appear to be stark differences in gene expression between all dose levels. On the other hand, most of the response due to exposure to FC occurs under a high dose level, the differences between low dose and no treatment having a much smaller representation. For SiO_2 _and SWNT, most of the difference is between high or low dose (it does not matter) and no treatment. These observations are consistent with what was inferred from the heatmaps and MDS plots in Figures 1, 2, 3, and 4.

**Table 3:** Number of genes that have significant differences among the top 40 genes.

	HD-LD	HD-Cont.	LD-Cont.
CB	36	40	32
FC	40	40	7
SiO2	25	40	40
SWNT	27	40	39

## Conclusion

The analysis presented here lead to interesting and complementary conclusions about the response across time of human epidermal keratinocytes after exposure to nanomaterials. For example, we observed (especially through the time sequence of MDS plots) that gene expression for each treatment become closer to the expression of the baseline cultures as time proceeds, except for CB HD and FC HD, which generally have the most changing expression levels. The results from the MDS analysis mostly confirmed the results from the heatmaps and SOM clustering. The genes found to be differentially-expressed by the ANOVA analysis are involved in a number of cellular processes, including regulation of transcription and translation, protein localization, transport, cell cycle progression, cell migration, cytoskeletal reorganization, signal transduction, and development. The majority of these genes show significant differences at both the low and high dose levels.

## Supplementary information

http://www.ece.tamu.edu/~ulisses/HARC/index.html

## Competing interests

The authors declare that they have no competing interests.

## Authors' contributions

AZ: Bioinformatics, drafted the manuscript; For MJC: cell culture, coordinated RNA isolation and microarray processing, conceived the study, helped draft the manuscript; UBN: Bioinformatics, helped draft the manuscript; ERD: Participated in design and coordination and helped draft the manuscript.
